# Genome-wide identification and characterization of *COMT* gene family during the development of blueberry fruit

**DOI:** 10.1186/s12870-020-02767-9

**Published:** 2021-01-06

**Authors:** Yushan Liu, Yizhou Wang, Jiabo Pei, Yadong Li, Haiyue Sun

**Affiliations:** 1 Engineering Center of Genetic Breeding and Innovative Utilization of Small Fruits of Jilin Province, College of Horticulture, Jilin Agricultural University, Changchun, 130118 China; 2 College of Life Sciences, Jilin Agricultural University, Changchun, 130118 China; 3 Key Laboratory of Plant Resources/Beijing Botanical Garden, Institute of Botany, Chinese Academy of Sciences, Beijing, 100093 China; 4 University of Chinese Academy of Sciences, Beijing, 100049 China; 5 Institute of Horticulture, Hangzhou Academy of Agricultural Sciences, Hangzhou, 310000 China

**Keywords:** *Vaccinium corymbosum*, *O*-methyltransferase, evolution; fruit development

## Abstract

**Background:**

Caffeic acid *O*-methyltransferases (*COMTs*) play an important role in the diversification of natural products, especially in the phenylalanine metabolic pathway of plant. The content of *COMT* genes in blueberry and relationship between their expression patterns and the lignin content during fruit development have not clearly investigated by now.

**Results:**

Ninety-two *VcCOMTs* were identified in *Vaccinium corymbosum*. According to phylogenetic analyses, the 92 *VcCOMTs* were divided into 2 groups. The gene structure and conserved motifs within groups were similar which supported the reliability of the phylogenetic structure groupings. Dispersed duplication (DSD) and whole-genome duplication (WGD) were determined to be the major forces in *VcCOMTs* evolution. The results showed that the results of qRT-PCR and lignin content for 22 *VcCOMTs*, *VcCOMT40* and *VcCOMT92* were related to lignin content at different stages of fruit development of blueberry.

**Conclusion:**

We identified *COMT* gene family in blueberry, and performed comparative analyses of the phylogenetic relationships in the 15 species of land plant, and gene duplication patterns of *COMT* genes in 5 of the 15 species. We found 2 *VcCOMTs* were highly expressed and their relative contents were similar to the variation trend of lignin content during the development of blueberry fruit. These results provide a clue for further study on the roles of *VcCOMTs* in the development of blueberry fruit and could promisingly be foundations for breeding blueberry clutivals with higher fruit firmness and longer shelf life.

**Supplementary Information:**

The online version contains supplementary material available at 10.1186/s12870-020-02767-9.

## Background

Blueberries have become widely appreciated worldwide because they contain phytonutrients such as flavonoids, which were discovered in the early 1900s [1–4]. The flavonoids in blueberry fruits have been confirmed to control diabetes, exert anti-inflammatory and neuroprotective, effects and protect eye health through their antioxidant activity [5]. Because the functions of blueberry component have made it to be accepted by an increasing number of people as “super fruits” [6], global blueberry production has greatly grown 35% from 2004 to 2016 [7]. However, because of respiration, evaporation, pathogen infection and cell wall degradation, the blueberry fruits have a characteristic of high perishability [8]. How to maintain the quality of flesh blueberry fruit is an urgent problem.

Major thrusts of research on the blueberry fruit softening are in two ways. One is on the mechanism of fruit softening related to cell wall structure and some hydrolytic enzyme [9, 10], the other one is to extend shelf life by external treatment like cold stage [11], high oxygen treatment [12], cuticular wax preservation [13], ethylene absorbent treatment [14], sodium nitroprusside treatment [15] and acibenzolar-S-methyl treatment [8]. The main theory of sodium nitroprusside treatment and acibenzolar-S-methyl treatment is to improve the activities of phenylalanine ammonia lyase (PAL) and CoA ligase (4CL) in lignin metabolism pathway and Peroxidase (POD) to catalyze the polymerization of precursors of phenolic substances into lignin phenols, so as to make the fruit lignified, strengthen the host cell wall and inhibit pathogen growth [16].

Lignin is a characteristic component of cell walls. Treatment of fruits can induce changes in the lignin biosynthesis pathway to influence the metabolites to have an effect on the pathogen infection and fruit firmness [17]. At present, many fruit trees and vegetables have been reported their effect of lignification on postharvest fruits, such as strawberry [18], red raspberry [19], zucchini fruit [20] and blueberry [15]. The main treatment methods of affecting lignification are external application after harvest. There are only a few studies on genetic modification to increase fruit lignification to make the preservation period prolonged effectively.

*O*-methyltransferases (OMTs) are a multifunctional enzyme in the lignin and flavonoid biosynthesis pathway, in *Arabidopsis thaliana* it can converse caffeic acid to ferulic acid and 5-OH coniferaldehyde/5-OH coniferyl alcohol to sinapaldehyde/sinapyl alcohol, forming G and S units of lignin [21]. COMTs catalyze N-acetyl serotonin into melatonin [22, 23]. The overexpression of them also can help plant grow [24]. *Sorghum bicolor* COMT can be involved in tricin biosynthesis methylated the flavones luteolin and selgin [25]. The expression of *MOMT4* in aspen can change the structure of lignin, which increase the crosslinking of condensed lignin subunits by G-units [26]. On the flavonoid biosynthesis pathway, the antioxidant activity of flavonoids is related to the number of hydroxyl substituents: greater numbers of hydroxyl substituents are associated with stronger antioxidant and prooxidant activities. O-methylation of hydroxyl substituents inactivates both the antioxidant and prooxidant activities of flavonoids [27]. OMTs can be divided into two groups: PI-OMT I family and PI-OMT II family [28]. PI-OMT I family forms by CCoAOMTs, and COMTs belongs to PI-OMT II family. Most of COMTs have two types of domain, Dimerisation (PF08100) and Methylransf_2 (PF00891). There are 7 motifs conserved in COMTs, among them motif A and motif E may be the putative SAM-binding domains. COMTs have a wider range of catalytic substrates such as lignin precursors, alkaloids, flavonoids [29]. These compounds play an important role in plant growth and development and in the face of biotic and abiotic stresses. Therefore, plant OMT enzymes have been widely studied [2, 30, 31].

Publications of different plant genomes has enabled analyses of COMT family genes in several species to be carried out [32, 33]. Blueberry has been widely studied because of its large amounts of flavonoids. The tetraploid blueberry genome was released in 2019 [34]. In this study, we identified COMTs family to find OMTs that may related to the methylation of lignin precursors and flavonoids during the growth and development of blueberry fruits Based on the genome of tetraploid blueberry. The results of this study will build foundations for breeding blueberry cultivars with higher fruit firmness and longer shelf life.

## Results

### Phylogenetic and sequence analyses of *COMT* genes in blueberry

To identify *COMT* genes in the blueberry genome, one characterized sequence from *Arabidopsis thaliana* (AT5G54160) and 36 identified sequences from *Populus trichoarpa* were used as a set of queries in a BLASTP search (E < 1e-5) [35]. In all, 123 candidate sequences were retrieved from the blueberry genome. Then, all the 123 candidate sequences scanned for a Methyltransf_2 domain. Ninety-two sequences with a Methyltransf_2 domain were identified in blueberry. All of them were mapped to pseudochromosomes (VaccDscaff1-VaccDscaff48) and renamed from VcCOMT1 to VcCOMT92 according to orders of location on the pseudochromosomes. Gene characteristics were analyzed in Table S1 (Additional file 1: Table S1). The result showed that VcCOMT56 was the shortest protein (112 amino acid) and the longest one was VcCOMT89. The analysis of molecular weight showed that 92 VcCOMT proteins ranged from 12 to 201 kDa, and the isoelectric point ranged from 4.62 to 8.73.

**Fig. 1:**
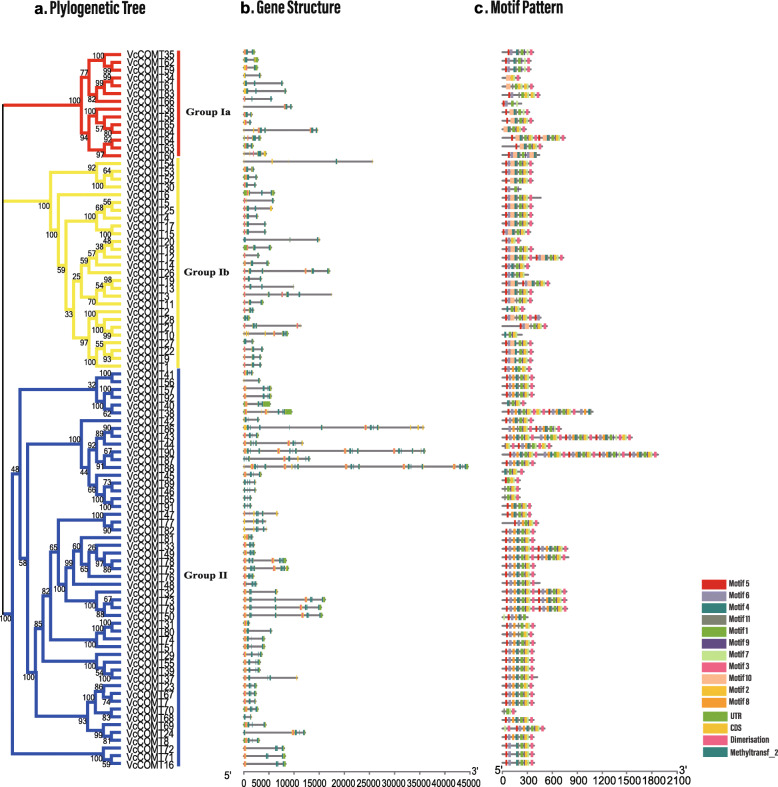
The polygenetic relationship, gene structure and motif analysis of the *VcCOMTs* from blueberry. **a** The phylogenetic tree was constructed by MEGA7.0 with the ML method. **b** Structures of the 92 putative *VcCOMT* genes. **c** Motif distribution of VcOMTs proteins. The different motifs are indicated by different colors for motifs 1–11, and the sequences of 11 motifs were in the Additional file 2: Fig. S1

A maximum likelihood (ML) phylogenetic tree created by using blueberry COMT protein sequences showed that the sequences were distributed into 2 groups, and this finding was supported by high bootstrap values and gene structure (Fig. 1a). Gene structure and conserved domain analysis revealed that all COMTs had a C-terminal catalytic domain named Methyltransf_2 domain including a SAM/SAH binding pocket and a substrate-binding site. Some of them showed a common structure with an N-terminal domain called Dimerization [36]. The SAM/SAH binding pocket was highly conserved, while the substrate binding sites were specific to proteins in different groups [37]. The domains of the COMTs in the same group had similar quantities and sizes of introns (Fig. 1b). For example, one Dimerization domain in all the groups was on the one exon. This situation of gene structure was different from Methyltransf_2 domain. In the Group Ia and Group Ib, VcCOMTs had Methyltransf_2 domain distributed by two exons which had one intron in the middle except VcCOMT6, VcCOMT61 and VcCOMT83. They had the Methyltransf_2 domian distributed on three exons with two introns. Although the Methyltransf_2 domain also distributed on three exons with two introns in the Group II, the structure of domain was different from VcCOMT6, VcCOMT61 and VcCOMT83. The second exon in the Group II was very small. Different from the reported *Populus trichoarpa* that COMTs has only one Methyltransf_2 domain in one sequence, some blueberry COMTs had two or three Methyltransf_2 domains in one sequence [38]. However, the gene structure of Methyltransf_2 domain in VcCOMTs was similar in sequences in the same group. The differences in protein sequences among the blueberry COMTs were analyzed by using Multiple Expectation Maximization for Motif Elicitation (MEME) online tools. In all, 11 motifs were found in the blueberry COMT sequences [35]. Most of the motifs were same in two groups and they were in the same order in COMT sequences within the same group (Fig. 1c). Motifs 10 was special to Group I and only Group II had motif 8. The similar genetic structures and conserved motifs within groups supported the reliability of the phylogenetic structure groupings.

### The Tandem (TD) events and collinearity analysis of *VcCOMTs*

**Fig. 2:**
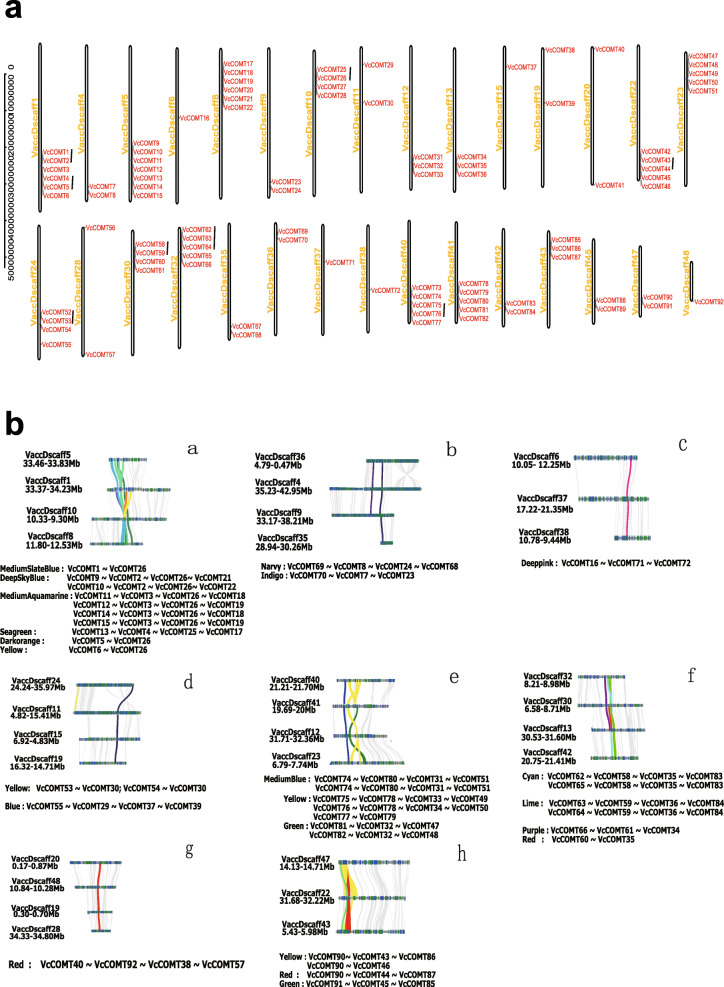
The location of *VcCOMTs* on the pseudochromosomes and the collinearity of *VcCOMTs* between the homologous chromosome. **a** The location of *VcCOMTs* on the Pseudochromosome. **b** The collinearity of *VcCOMTs* between the homologous chromosome, same color between different homologous chromosome was representative the collinearity of *VcCOMTs*

According to previous studies, a chromosomal region 150–200 kb in length that contains two or more genes is evidence of a tandem [33]. Nine pairs of tandem gene pairs were found in the blueberry genome by MCscanX (*VcCOMT1*/*VcCOMT2, VcCOMT4/VcCOMT5, VcCOMT25/VcCOMT26, VcCOMT43/VcCOMT44, VcCOMT52/VcCOMT53, VcCOMT58/VcCOMT59, VcCOMT62/VcCOMT63, VcCOMT63/VcCOMT64, VcCOMT75/VcCOMT76*). Ninety-two *COMTs* were mapped to the 48 chromosomes exhibited evidence of 9 TD events on blueberry pseudochromosomes (Fig. 2a) [39]. Ninety-two *COMTs* allowing for the detected of 83 collinear relationship (Fig. 2b). The line of same colour between two *COMT* genes on the chromosomes indicates collinearity. The collinearity of *VcCOMTs* among the different homologous chromosomes existed in different forms. The first form was one *VcCOMT* on the one chromosome while to the other *VcCOMT* was on the other chromosome just like group b, c, d, g (Fig. 2b). The other was one *VcCOMT* on the one chromosome to some *VcCOMTs* on the other chromosome just like *VcCOMT11*, *VcCOMT12*, *VcCOMT14*, *VcCOMT15* had a collinearity to the *VcCOMT3*, respectively. This reasons for this phenomenon might be attributed to its allopolyploid genome [34]. Most of the events were located in highly duplicated blocks and were identified as WGD or segmental duplication events with MCScanX. This result indicated that the *VcCOMT* gene family has expanded and evolved through genome-wide duplication.

### Analysis of *VcCOMT* gene promoters in blueberry

**Fig. 3:**
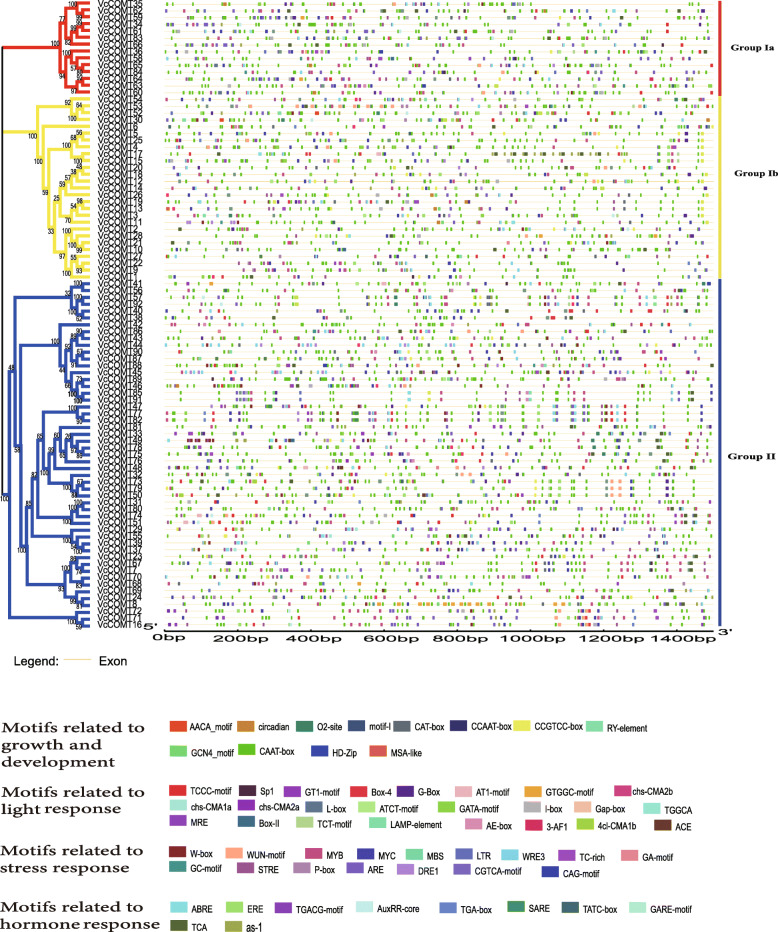
Predicted cis-elements in the promoter regions of *VcCOMT* genes

The start of transcription is a key stage of gene expression, and an important event in this stage is the interaction between RNA polymerase and the promoter. The structure of the promoter affects the binding affinity of RNA polymerase, thus affecting the level of gene expression [32]. We analyzed the *cis*-acting elements on blueberry *COMT* genes (Fig. 3). The results for the blueberry *COMTs* were similar to the results for *Catalpa bungei COMTs* [33]. According to the function, the *cis*-acting elements from *COMTs* could be divided into four classes. Light response-related motifs constituted the majority of the *cis*-acting elements on the blueberry *COMTs* and were distributed in all groups. This finding indicated that the *COMT* genes in blueberry may be controlled by light. Many *cis*-acting elements related to plant growth and development were found in the promoter region such as AACA motif and GCN4 motif related to the endosperm, RY-element related to seed-specific regulation, circadian which was a regulatory element involved in circadian control and MSA-like element related to cell cycle regulation. We found that there are some stress-related cis-regulatory elements (CREs) and some hormone related CREs in the promoter region of *COMTs* such as LTR, ARE, TC-rich repeats and others related to stress response, ABRE, ERE, TGA-BOX, TCA, as-1 which related to hormone. And MYB binding sites, MYC binging sites and W-box were also found in the promoter region which were transcription factor binding sites with MYB, bHLH and WRKY protein. The promoters of *VcCOMTs* within the same subgroup were similar. Often, the sequences with higher similarities and higher collinearity on the homologous chromosomes, the types and even orders of the *cis*-acting elements of them were similar, just like *VcCOMT59* and *VcCOMT64*, *VcCOMT34* and *VcCOMT66*, *VcCOMT60* and *VcCOMT65* in the Group Ia, the *VcCOMT26* and *VcCOMT13*, *VcCOMT22* and *VcCOMT9* in the Group Ib, the *VcCOMT77* and *VcCOMT82*, *VcCOMT78* and *VcCOMT75*, *VcCOMT16*, *VcCOMT71* and *VcCOMT72* in the Group II, especially within the paralogous pairs such as *VcCOMT57* and *VcCOMT92*, *VcCOMT85* and *VcCOMT91*, *VcCOMT31* and *VcCOMT80*, *VcCOMT37* and *VcCOMT39*. Similar regulatory elements within sequences may greatly influence similarities among gene expression patterns and gene functions. A large majority of *VcCOMTs* had ABRE, related to the abscisic acid and TCA motif related to the salicylic acid. The unique regulatory elements in different subgroups, may underlie the different functions of the genes in different subgroups, for example, GCN4, related to the endosperm, main distributed on *VcCOMTs* which were in Group Ib and Group II, while the circadian related to the circadian rhythm mainly distributed in Group Ia and Group Ib.

### Evolutionary analysis of *COMT* genes in blueberry and other species

**Fig. 4:**
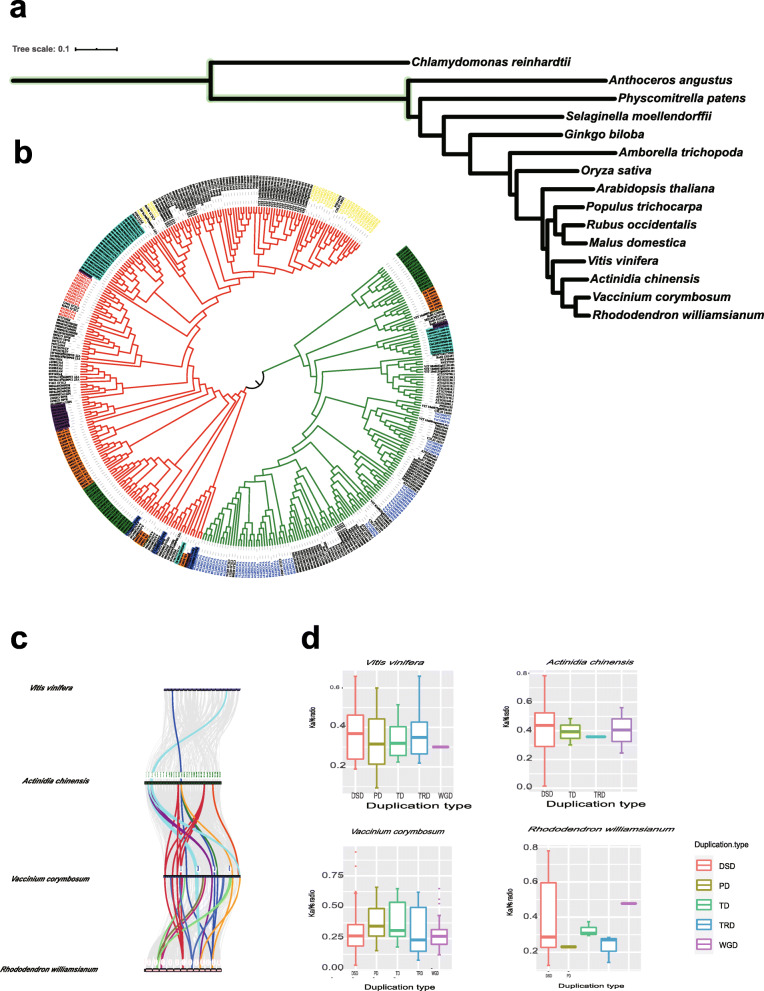
Evolution of COMTs in different plant species. **a** The evolution relationship of 15 plants in research. **b** A phylogenetic trees for *COMTs* from 15 plants. (The red, yellow and blue characters correspond to Group Ia, Group Ib, Group II, respectively; red - *Anthoceros angustus*, dark blue - *Physcomitrella patens*, green - *Selaginella moellendorffii*, orange - *Ginkgo biloba*, purple - *Amborella trichopoda*, sky blue - *Oryza sativa*; clade red – cluster I; clade green-cluster II). **c** Synteny analysis of *VcCOMT* genes between blueberry and three plant species. Gray lines in the background indicate the collinear blocks within blueberry and other plant genomes. The same color represents *COMTs* with collinearity in different genomes. **d** Ka/Ks ratio of *Vitis vinifera, Actinidia chinensis, Rhododendron williamsianum* and *Vaccinium corymbosum*

Four hundred twenty-five COMT sequences were identified in 16 plant genomes including one Chorolphyta, one Charophyte green algea (CGA) and 14 land plants by Hidden Markov Model (HMM) search (Fig. 4a). The CGA were the closest living relatives of land plants [40], but there was no putative COMT searched in *Chara braunii*. In the genome of green algae *Chlamydomonas reinhardtii*, three putative COMTs were identified in it and they did not have complete Methyltransf_2 domain. Two of them had other domain Dimerisation2 (PF16864.5) which was different from land plant COMTs*.* The progression from Dimerisation2 in algae to the Dimerization domain in land plants might suggests the evolution of the *COMTs* from algae to land plants. In the three more ancient genomes in our study, *Anthoceros angustus*, *Physcomitrella patens* and *Selaginella moellendorffii*, we identified 3, 7 and 34 putative genes, respectively. Compared with the early vascular plant *Selaginella moellendorffii,* the number of *COMTs* in the *Anthoceros angustus* and *Physcomitrella patens* which didn’t have vascular was 10 and 5 times higher than those in the *Anthoceros angustus and Physcomitrella patens,* respectively. The percentage of putative *COMTs* in the total number of genes as well as the number of *COMTs* per megabase of genome in *Selaginella moellendorffii* were found higher than in Bryophyta. They indicated that the expansion was not related necessarily to an increase in the genome size but could be determined by the development of new functions, the deposition of lignin and the existence of abundant flavonoids [41]. The number of *COMTs* in diploid apple and that in diploid grape was approximately half of that in tetraploid blueberry (Table 1). In the apple genomes, the percentage of putative *COMTs* was almost equal in the total number of genes with blueberry *VcCOMTs* while it was a two-fold decline in the grape genome. To study the evolutionary relationships of the *COMTs* in the land plants, candidate *COMTs* from 15 plant species, including *Chlamydomonas reinhardtii, Anthoceros angustus, Physcomitrella patens, Selaginella moellendorffii, Ginkgo biloba, Amborella trichopoda, Oryza sativa, Arabidopsis thaliana, Populus trichocarpa, Malus domestica, Rubus occidentalis, Vitis vinifera, Actinidia chinensis, Rhododendron williamsianum* and *Vaccinium corymbosum* were used to construct a phylogenetic tree, and the *COMTs* from the alga *Chlamydomonas reinhardtii* were used as outgroups (Fig. 4b). The phylogenetic analysis indicated that the *COMTs* were divided into two clusters. The cluster I was red which was contained *COMTs* from all the 14 land species. The cluster II (clade is green) didn’t have *COMTs* in the *Anthoceros angustus, Physcomitrella patens*, which indicating that they might be orthologous genes originating from a single ancestral gene but a new function of *COMTs* occurred from *Selaginella moellendorffii* and led to gene differentiation [49, 50]. *COMTs* in *Selaginella moellendorffii*, were not clustered together with those in angiosperms, and the gymnosperm species in cluster II. The results suggested that *COMT* had been recruited for S lignin biosynthesis independently in angiosperms, the gymnosperm and *Selaginella moellendorffii* [51].

**Table 1:** COMT genes in the different genomes sequenced

Plant species	Predicted number of genes	Putative COMTs retrieved	Putative COMTs	References	Genome size (Mb)
*Chlamydomonas reinhardtii*	19,528	6	3	Ensembl plant	112
*Anthoceros angustus*	14,269	8	7	[42]	119
*Physcomitrella patens*	86,669	14	4	Ensembl plant	480
*Selaginella moellendorffii*	34,825	46	34	Ensembl plant	212.5
*Ginkgo biloba*	41,480	52	46	[43]	10,864.64
*Amborella trichopoda*	27,313	20	14	[44]	706
*Oryza sativa*	42,355	36	34	Ensembl plant	389
*Arabidopsis thaliana*	48,321	29	17	Ensembl plant	135
*Populus trichocarpa*	73,012	60	40	Ensembl plant	485
*Malus domestica*	40,624	62	48	Ensembl plant	750
*Rubus occidentalis*	33,286	61	17	[45]	293
*Vitis vinifera*	29,927	51	48	[46]	487
*Actinidia chinensis*	33,115	15	10	[47]	758
*Rhododendron williamsianum*	21,419	22	14	[48]	491.6
*Vaccinium corymbosum*	118,456	123	92	[34]	1669.12

### The collinearity analysis, gene duplication events and Ka/Ks analysis of *COMTs* in blueberry and other plant species

To infer the evolutionary mechanism of *COMT* genes in tetraploid blueberry, we analyzed the collinearity among *Vitis vinifera* which indicated a palaeo-hexaploid ancestral genome for many dicotyledonous plants [46], *Actinida chinensis* which belongs to the Actindiaceae family in Ericales [52], an early divergent lineage within asterids and *Rhododendron williamsianum* which represented species-rich groups within Ericaceae [48] and *Vacciniun corymbosum* (Fig. 4c). The *COMTs* on homoeologous chromosomes that showed collinearity are indicated in the same colour in different plants. Two *COMTs* in the *Actinida chinensis* had one orthologous region in *Vitis vinifera*. One *COMT* in the *Actinida chinensis* had two orthologous regions in *Vitis vinifera*. These genes indicated that these orthologous pairs may have already existed before the ancient paleohexaplodiy (γ) event. *COMTs* of *Actinida chinensis* and *Vacciniun corymbosum* had higher collinearity. Most types of corresponding relationship of collinearity between *COMTs* in the *Actinida chinensis* and *Vaccinium corymbosum* were two *COMTs* in the *Actinida chinensis* to one *COMT* in the *Vaccinium corymbosum*. Some of corresponding relationship of collinearity between *COMTs* in two genomes were one *COMTs* to one *COMTs* in different genome indicating that some *COMTs* were lost during evolution. One *COMT* in the *Actinida chinensis* that had collinearity only with *Vaccinium corymbosum* among the other species, as shown in orange. These *COMTs* might have similar function. Interestingly, *COMTs* in *Rhododendron williamsianum* had highest collinearity with *COMTs* in *Vaccinium corymbosum*. The types between them were more complex, at most appeared 8 *COMT*s in *Vaccinium corymbosum* who had collinearity with one *COMTs* in the *Rhododendron williamsianum.*

*COMT* duplicated gene pairs were identified in four plants with *DupGen_finder* software. There were five categories of duplicated gene pairs, including WGD, TD, proximal duplication (PD), transposed duplication (TRD), and DSD pairs. Among the categories, the DSD category had the most duplicated gene pairs from the four plant species. In blueberry, the percentage of gene pairs derived from WGD was higher than the percentages of gene pairs derived from other processes. Grape had nearly the same numbers of PD-, TD-, and TRD-derived gene pairs. These three categories of events might have played almost the same roles in the evolution of grape. The pattern for azalea was the similar as that for grape. In addition, DSDs played a major role in the evolution of azalea, and TDs and TRDs might have played similar evolutionary roles. The DSDs and WGDs were the major drivers of evolution in blueberry and kiwi fruit. The Ks values between the homologous genes were used to estimate the time of divergence of the diploid progenitors from their most recent common ancestor (MRCA), which was determined to be between approximately 0.94 and 1.02 million years ago. According to the eq. T = Ks/2λ (λ, synonymous substitution rate; λ = 1.3e-8) [34], 42 *COMT* pairs were derived from WGD in blueberry before the estimated time of divergence of the diploid progenitors from their MRCA, while 4 were derived after that. The selection pressures on the *COMTs* in the four plant species were explored based on the Ka/Ks ratios. A Ka/Ks ratio greater than 1 indicated positive selection, a Ka/Ks ratio equal to 1 indicated neutral evolution, and a Ka/Ks ratio less than 1 indicated purifying selection at a low evolutionary rate. The Ka/Ks values of the *COMT* pairs in the four plant species were all less than 1 (Fig. 4d).

### Gene expression analyses with differential expression COMTs in blueberry fruits

**Fig. 5:**
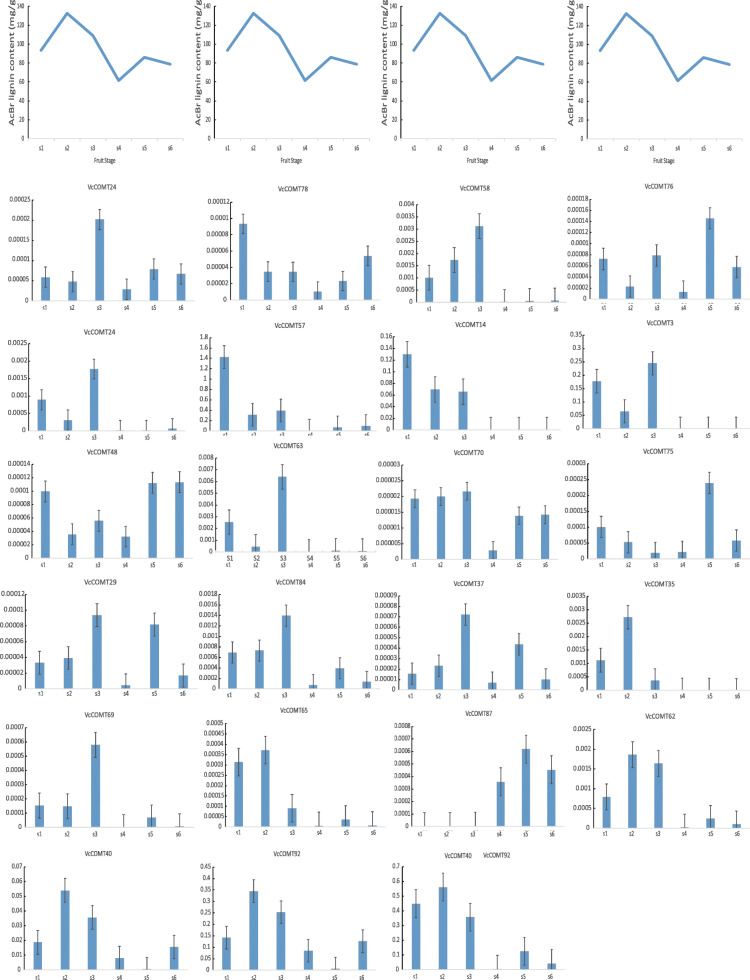
The lignin content and relative quantification of *VcCOMTs* during s1-s6 fruit development. The first line is the broken line chart of lignin content, the relative content of lignin in vertical coordinate, and the abscissa of different fruit development stages; The rest were 22 *VcCOMTs* relative quantitative histogram, abscissa was different fruit development period, ordinate was relative content of genes

**Fig. 6:**
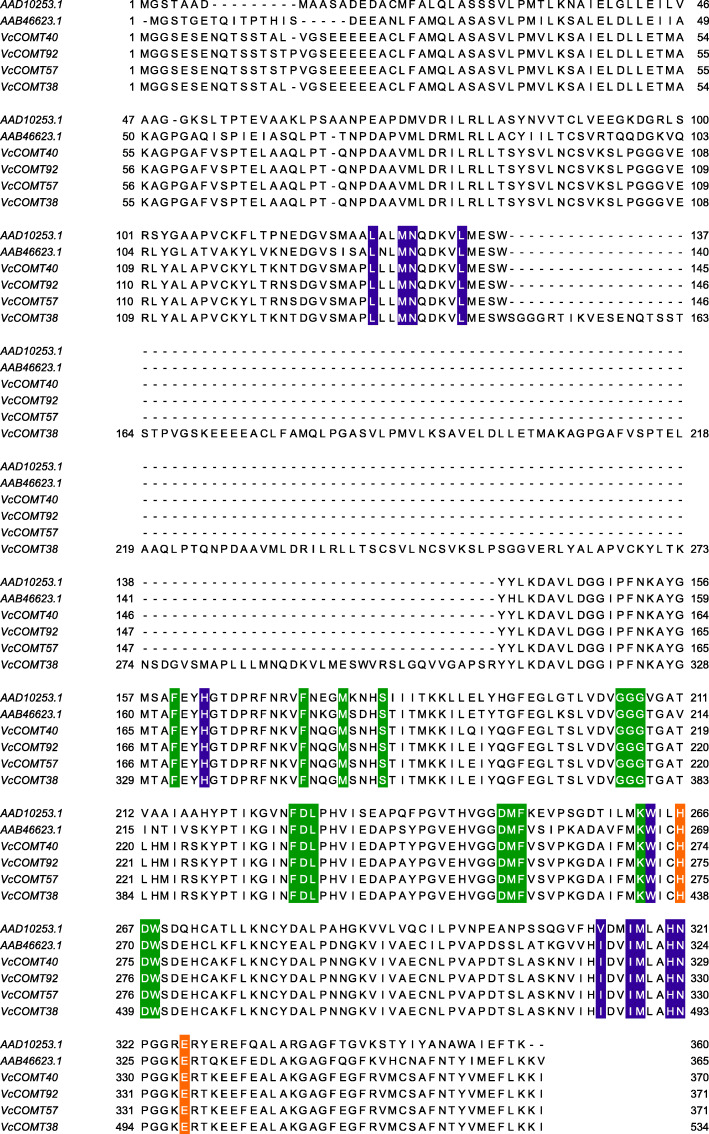
*VcCOMT40*, *VcCOMT92* Multiple sequence alignment was performed with other related to lignin *COMT*. Green: SAM binding; Blue: Substrate binding; Orange: catalytic residues

Twenty-two *VcCOMTs* that were differentially expressed during fruit development according to their expression in the transcriptome analysis (|log2(fold change, FC)| > 1, *P* value < 0.05) were selected for qRT-PCR at different fruit development stages. Based on the lignin content, we selected three genes related to lignin changes during fruit development, *VcCOMT62, VcCOMT40* and *VcCOMT92* (Fig. 5, Additional file 4: Table S3). The expression trends of *VcCOMTs* and the content variation trends of lignin in the early time were similar, which increased in s1 to s2 and then decreased. The s2 was the highest point. The trend of *VcCOMT62* was consistent with that of lignin during the fruit development, but the relative expression content was very low. The relative content of *VcCOMT40* and *VcCOMT92* was relatively high in fruit development stage. The lowest expression of *VcCOMT40* and *VcCOMT92* were different from the lignin in the lowest lignin content during the fruit development. *VcCOMT40* and *VcCOMT92* were on the homologous chromosomes which had high sequence similarity in the gene collinearity region. After designing a pair of primers in the collinear region between *VcCOMT40* and *VcCOMT92*, the expression trend was consistent with that of lignin during the fruit development stage. According to the results of multiple sequence alignment (Fig. 6), *VcOMT40 and VcCOMT92* contained the same substrate binding sites with COMT who could catalytic caffeic acid and 5-OH coniferaldehyde [37].

## Discussion

*COMTs* could react to various substrates, such as phenylpropanoids, flavonoids, and alkaloids; thus, they were ubiquitous in plants because of their importance in plants adaptation to the environment and to adversity [30, 53]. As long ago as in the last century, scientists began to be interested in the roles of *COMT* genes in plants [54, 55]. The publication of different plant genomes had enabled analyses of *COMT* family genes in several species to be carried out [38, 56, 57]. Blueberry had been widely studied because of its large amounts of flavonoids. The tetraploid blueberry genome was released in 2019, and 92 *COMTs* have been identified, named VcCOMT1-VcCOMT92 based on their chromosome positions. According to phylogenetic and gene structure analyses, these 92 *COMT* genes could be divided into 2 groups, named Group Ia, Group Ib and Group II. The sequences and structural similarities were greater within the same branch than between branches. Based on analysis of the conserved motifs, the three groups of *COMTs* can be roughly divided into two categories [20]. Among the Group Ia and Group Ib all contain motif 10, while the other groups do not. Motif 10 is approximately 15–50 amino acids upstream of the VcCOMT sequence and forms the back wall of the binding pocket [36, 37, 57, 58]. Perhaps because of the different binding substrates, the VcCOMT sequences of the two categories are different from each other. We identified these motifs which were highly conserved in COMTs. Some residues in four motifs (motif I: DVGGG, motif II: DLPHV, motif III: GDMF, and motif IV: VPKGDAIFLKWI) are related to the SAM/SAH binding site [58]. Motif 2 of the VcCOMTs contained motif I (DVGGG) and some of motif II (DLPHV). Motif 1 of the VcCOMTs contained motif III (GDMF) and motif IV (VPKGDAIFLKWI) (Additional file 2: Fig.S1) [28]. Gene duplication probably contributes to the evolution of species and to the adaptation of species to their environments [59]. In the blueberry genome, candidate *VcCOMTs* were analyzed according to the collinearity of homoeologous chromosomes with MCscanX [60]. The numbers of *VcCOMTs* with collinearity differed on different chromosomes (Fig. 2b). The many-to-one ratio may exist because some copies of *COMT* in different chromosomes have been lost due to the influence of the environment during the evolution of blueberry or because some redundant genes with incomplete domains are present. The one-to-many ratio may be a result of distinct subfunctionalization and neofunctionalization. Two *COMTs* sequences with collinearity and high sequence similarity on homologous chromosomes had similar promoter sequence in the blueberry genome. The cis-regulatory elements present in the promoter regions were the binding sites of *COMTs* gene with other proteins to play a central role in regulating gene transcription. There were a large number of light response related regulatory elements, rhythm elements and regulatory elements that promote plant endosperm and seed growth, which may be related to plant growth and lignin synthesis [61, 62]. In the promoter region of the *COMT* genes of blueberry, some regulatory elements related to hormones and stress were also found, which was consistent with previous studies. When plants were stressed or treated with external hormones, the content of *COMTs* increased [63–66].

In this study, different numbers of *COMT*s were identified in 15 plant species ranging from algae to land plants (Table 1). The evolution of *COMTs* from algae to land plants led to a change in the Dimerization domain (Additional file 1: Table S2, Additional file 3: Fig.S2). Furthermore, we found that the number of COMTs in *Selaginella moellendorffii* was greater than the numbers in other dicotyledonous species and less than the numbers in *Vitis vinifera*, *Malus X domestica* and *Vaccinium corymbosum*. The development of vascular tissues underlies the differences between *Selaginella moellendorffii* and Bryophytes. Lignin is the main component of vascular tissue and provides plants with structural support to stand upright. *COMTs* are important methyltransferases in lignin biosynthesis that methylate components of lignin similar to the S units in *Selaginella moellendorffii* [51]. The present research suggests that the evolution of lignin in land plants correlates with the evolution of *COMT* genes [38].

Comparison of the collinearity of the *VcCOMTs* in blueberry with the *COMTs* in the other plant species showed that the *VcCOMTs* that had collinearity with other *COMTs* were almost the same for the different species. Some *COMT* collinearity gene pairs between blueberry and kiwi fruit exhibited form of one *COMT* gene in blueberry to two *COMT* genes in kiwi fruit, but the collinearity pairs between blueberry and azalea exhibited one-to-many form. Perhaps the results indicated that kiwi fruit has undergone two rounds of WGD [39, 47]. And form indicates that *COMT* genes were duplicated after the differentiation of *Vaccinium corymbosum* and *Rhododendron williamsianum.* Gene duplication has five forms: DSD, PD, TRD, TD, and WGD [39]. Different gene replication patterns have different effects on the expansion of the COMT family in different plant species. DSD was the main feature of evolution in the four plant species except grape. Previous studies have revealed that the *COM*T genes all have tandem duplicates on all of the homoeologous chromosomes [34]. In the current study, TD of *VcCOMTs* was not identified on all of the homoeologous chromosomes by MCscanX. Fewer *VcCOMTs* arose through TD than through WGD. However, amplification of *COMT* genes in the blueberry genome occurred mainly through DSD and WGD. In contrast, the main drivers of gene expansion are WGD and TD in *Populus* [38]. In citrus, the numbers of TD and WGD events are similar [35]. *COMTs* have similar gene copy numbers in maize, rice and foxtail millet, and gene expansions in these genomes are mainly generated by TD and segmental duplication [32]. The WGD Ks of kiwi fruit *COMTs* is less than the Ad-β mean Ks of *Actinidia chinensis*. This result suggests that the WGD of kiwi fruit *COMTs* occurred before the shared WGD of Ad-β. The WGD Ks of tetraploid blueberry *COMTs* is also less than the Ad-β mean Ks of diploid blueberry. This result suggests that the WGD of tetraploid blueberry *VcCOMTs* occurred before the shared Ericales WGD Ad-β event. The WGD Ks of *Rhododendron williamsianum COMTs* is between the Ks of the Ad-β event and the Ks of the At-γ event. This suggests that the WGD of *Rhododendron williamsianum* occurred between two shared events. The Ka/Ks ratios of the five gene replication patterns of the *COMTs* from the four plant species were less than 1, indicating that the *COMTs* have experienced strong purifying selection [48].

During fruit development, the content of lignin in fruit increased first and then decreased. This phenomenon may be related to the formation of lignin during fruit development. In the early stage, the fruit swells and hardens, and the lignin content becomes high. From the green fruit stage to the colour-turning stage, the fruit becomes soft, and the lignin content shows a downward trend [67]. Based on the *VcCOMT* differential expression data from RNA-seq, 22 *VcCOMTs* were selected for detection of gene expression using qRT-PCR. Three genes had similar trend as lignin expression during fruit development. Although *VcCOMT62* had same trend as lignin expression during fruit development. The relative expression of it during the fruit development was too low. It indicated that it was not a main gene to related to lignin content during fruit development. The relative expression of *VcCOMT40* and *VcCOMT92* during the fruit development was almost highest among all the *VcCOMTs*. But the expression trend of single gene was slightly different from that of lignin during the fruit development. Because of the high similarity of sequence, in order to reflected the role of individual genes, primers were designed where most of their sequences are different. We designed a pair of primers in the homologous region, including four *VcCOMT* genes (*VcCOMT38, VcCOMT57, VcCOMT40, VcCOMT92*) with very high similarity. When we performed qRT-PCR again, it found that the trend was consistent with that of lignin during fruit development. It is suggested that more than one gene is responsible for the biosynthesis of lignin content.

## Conclusions

Here, we identified 92 *COMT* genes from blueberry and 425 *COMT* genes from 15 other species. According to phylogenetic analysis of *COMTs*, we divided the *COMTs* into two groups, which indicated the existence of two ancestor genes. DSD and WGD were revealed to be the major forces of blueberry evolution. The Ka/Ks ratios of the gene duplication patterns for the *COMTs* from the four plant species were less than 1, indicating that the *COMTs* have experienced strong purifying selection. According to the qRT-PCR results for 22 *VcCOMTs*, *VcCOMT40, VcCOMT92* were highly expressed and may play important roles in the synthesis of lignin of blueberry fruit. The results of this study will build foundations for breeding blueberry cultivars with higher fruit firmness and longer shelf life.

## Methods

### Plant materials

**Fig. 7:**
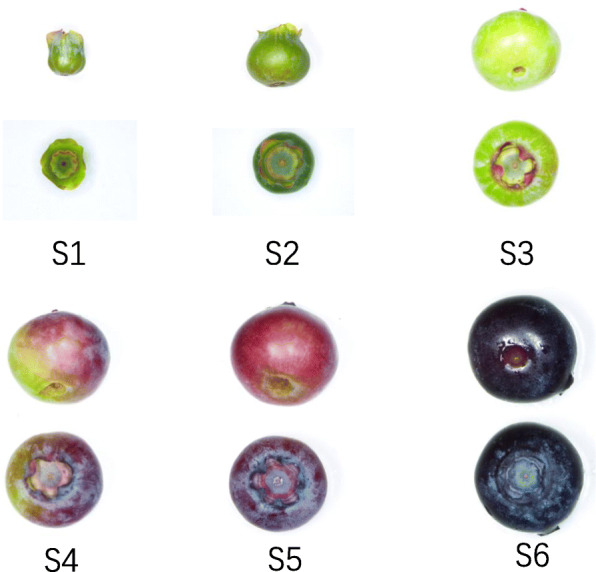
The stage of blueberry development naming s1 – s6

The samples were fruits of ‘Northland’ blueberry plants at 6 stages of growth and development that were obtained from the blueberry germplasm resource garden of Jilin Agricultural University. Stages 1 to 3 were sorted by increasing size (stage 1, 2–3.5 mm in diameter; stage 2, 4–7 mm; stage 3, 7–9 mm). Stages 3 to 6 were sorted by fruit color (stage 3, white blue, stage 4, 25–50% red skin; stage 5, predominantly purple skin with some red; stage 6, entirely dark blue and soft texture) [67](Fig. 7). The samples were taken from three different robust trees, frozen in liquid nitrogen and stored at − 80 °C.

### Identification of *COMT* genes in the genomes of blueberry and other plants

The graft blueberry genome was downloaded from the CoGe genome database (https://genomevolution.org/coge/SearchResults.pl?s=Vaccinium&p=genome). To identify complete *COMT* genes in the blueberry genome, one characterized sequence from *Arabidopsis thaliana* (AT5G54160) and 36 identified sequences from Populus trichoarpa were used as a set of queries in a BLASTP search (E < 1e-5). All the searched sequences were scanned for a specific domain (PF00891) with HMM in Pfam (http://pfam.xfam.org). Then, each possible sequence was analysed with the online program CD-search (https://www.ncbi.nlm.nih.gov/Structure/bwrpsb/bwrpsb.cgi) to identify the complete domains. We further identified COMT sequences in *Chlamydomonas reinhardtii, Anthoceros angustus, Physcomitrella patens, Selaginella moellendorffii, Ginkgo biloba, Amborella trichopoda, Oryza sativa, Arabidopsis thaliana, Populus trichocarpa, Malus domestica, Rubus occidentalis, Vitis vinifera, Actinidia chinensis, Rhododendron williamsianum* by HMM search*.*

### Phylogenetic, domain motif and gene structure analyses for the predicted *VcOMT* genes

First, the protein sequences of VcCOMTs from blueberry and other species were subjected to multiple sequence alignment and ML methods with 1000 bootstrap replicates in MEGA 7.0. The domain sequences of VcCOMTs from blueberry were predicted with CD-search. TBtools was used to perform exon/intron structure analysis for the *VcCOMT* genes (https://github.com/CJ-Chen/TBtools) with the mRNA sequences and genomic sequences [68]. The MEME suite (http://meme-suite.org/tools/meme) was used to analyze the motifs of VcOMT sequences with the following parameter setting: out motifs, 11.

### Analysis of collinearity between *COMTs* from blueberry and *COMT*s from other species

Collinearity analysis of *VcCOMTs* was performed with MCScanX (https://github.com/tanghaibao/jcvi/wiki/MCscan-(Python-version). Software was used to analyse the collinearity of *COMTs* between kiwi fruit and grape, blueberry and azalea, and blueberry and kiwi fruit.

### Analysis of *COMT* gene promoters in blueberry

The elements in the promoter fragments of the *VcCOMT* genes (1500 bp upstream of the translation initiation sites) were identified using the online program PlantCARE (http://bioinformatics.psb.ugent.be/webtools/plantcare/html/).

### Gene duplication and calculation Ka and Ks with *COMTs* from four species

The gene duplication from blueberry, grape, azalea and kiwi fruit was by *DupGen_finder* (https://github.com/qiao-xin/DupGen_finder), and Ka, Ks and the Ka/Ks ratio were calculated using the KaKs_Caculator by GLWL model. Therefore, a *P*-value < 0.05 was retained.

### Expression analysis of *VcCOMTs* in blueberry by qRT-PCR

Twenty-two *VcOMTs* were selected for qRT-PCR. The primers for the genes were designed using Primer Premier 5.0. Total RNA was isolated from s1- s6 fruits by the CTAB isolation method. The RNA was checked from a 1.2% agarose gel under UV light with no smearing before concentration detection by spectrophotometry. One microgram of total RNA was used to synthesize cDNA with a PrimeScript™ RT Reagent Kit with gDNA Eraser (TaKaRa, Japan) following the manufacturer’s instructions. The detailed methods of the experiment followed the instructions for SYBR Premix Ex Tag (Tli Rnase H Plus). *VcOMT* genes expression were analyzed in an ABI StepOnePlus Real-Time Quantitative PCR System (Applied Biosystems, Foster City, CA, USA). The thermos cycling parameters were the same as those used by Chen [69]. The *EIF* gene of blueberry was amplified with *EIFF* and *EIFR* primers (Additional file 1: Table S2) and used as a control to normalize the expression of the *VcOMTs* [70]. The real-time amplification data were analyzed by the Chen method, and a 40-cycle melting curve analysis was performed to ensure the reliability of the expression results. The results are expressed as the normalized relative expression levels (2^−ΔCT^) of the genes in various samples [69]. All experiments were run in triplicate.

### Analysis of lignin content

Acetyl bromide soluble lignin was determined in triplicate following the procedures described in [71]. Reference substance was Lignin (Dealkaline) (CAS: 900–53-2, Aladdin, China).

## Supplementary Information

**Additional file 1: Table S1.** Gene characteristics of *VcCOMTs* in blueberry genome. **Table S2.** Specific primers of rapeseed *VcCOMTs* used in qRT-PCR assays.

**Additional file 2: Figure S1.** The motif sequences of *VcCOMTs* (My own).

**Additional file 3: Figure S2.** The difference of algae and land plants COMT domain (My own).

**Additional file 4: Table S3.** Lignin content of fruit development.

## Data Availability

The blueberry genome was download from the CoGe genome database (https://genomevolution.org/coge/). The gene sequences of *Chlamydomonas reinhardtii, Galdieria sulphuraria, Physcomitrella patens, Selaginella moellendorffii, Amborella trichopoda, Vitis vinifera* were from Ensembl plants (https://plants.ensembl.org/index.html). The gene sequences of *Malus X domestica, Rubus occidentalis* were from GDR (http://www.rosaceae.org). The gene sequences of *Actinidia chinensis* were from PGDD (http://chibba.agtec.uga.edu/duplication/). The gene sequences of *Rhododendron williamsianum* was download from the CoGe genome database (https://genomevolution.org/coge/).

## Abbreviations

OMT: *O*-methyltransferases
COMT: Caffeic acid *O*-methyltransferases
DSD: Dispersed duplication
PD: Proximal duplication
TRD: Transposed duplication
TD: Tandem duplication
WGD: Whole-genome duplication
MRCA: Most recent common ancestor
