# Supplementary figures and images for: Genome-wide identification and characterization of COMT gene family during the development of blueberry fruit
Source: BMC Plant Biol. 2021 Jan 6;21:5. doi: 10.1186/s12870-020-02767-9 (PMC7789564; doi:10.1186/s12870-020-02767-9)

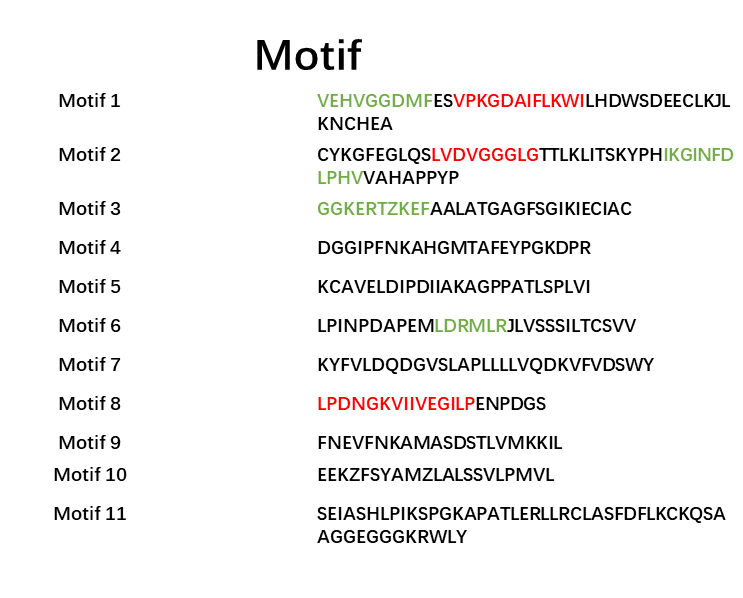

Supplement: Supplementary file 2 — Additional file 2: Figure S1. The motif sequences of VcCOMTs (My own). [file 12870_2020_2767_MOESM2_ESM.tif]

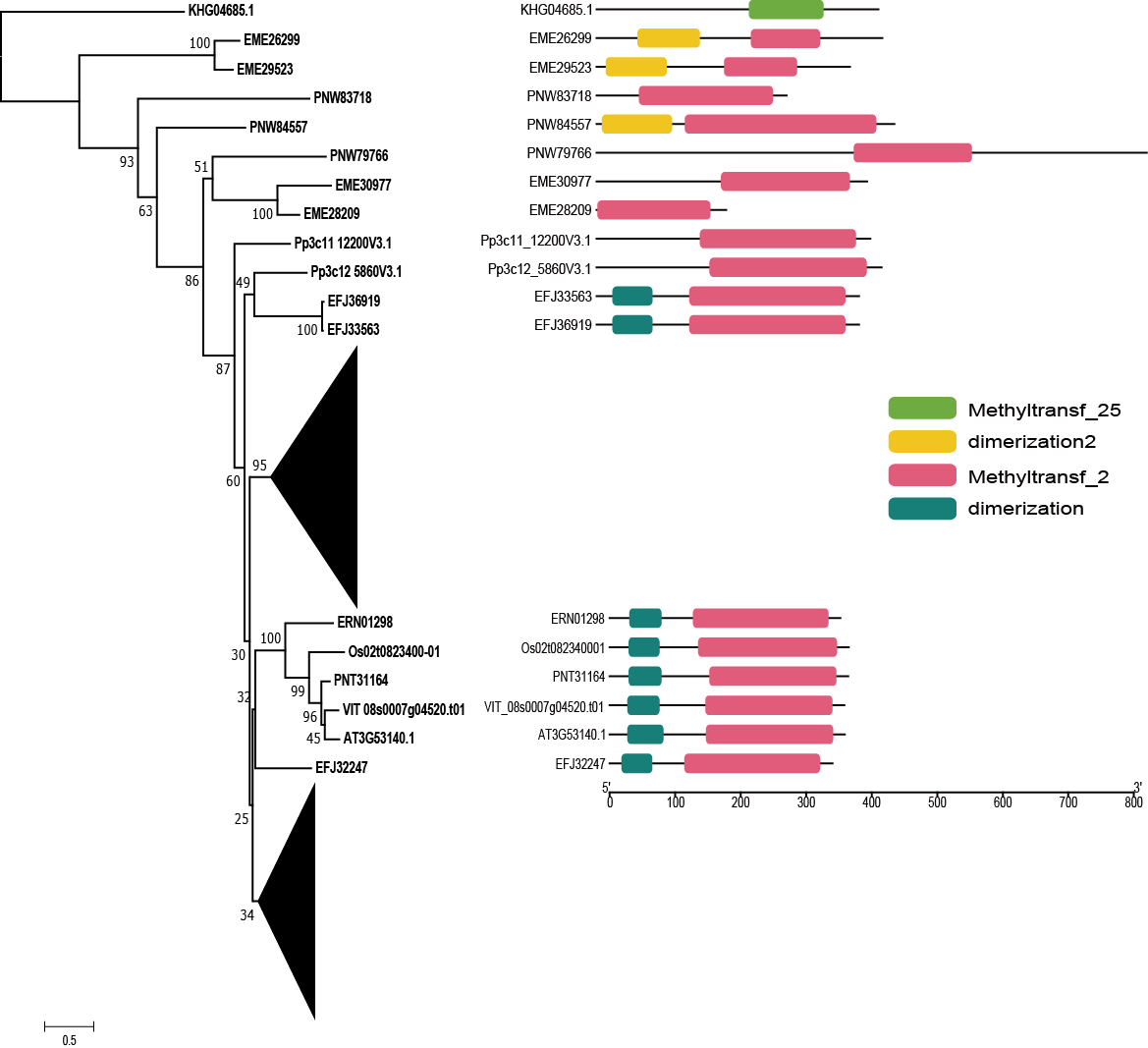

Supplement: Supplementary file 3 — Additional file 3: Figure S2. The difference of algae and land plants COMT domain (My own). [file 12870_2020_2767_MOESM3_ESM.png]
